# High-quality and universal empirical atomic charges for chemoinformatics applications

**DOI:** 10.1186/s13321-015-0107-1

**Published:** 2015-12-02

**Authors:** Stanislav Geidl, Tomáš Bouchal, Tomáš Raček, Radka Svobodová Vařeková, Václav Hejret, Aleš Křenek, Ruben Abagyan, Jaroslav Koča

**Affiliations:** National Centre for Biomolecular Research, Faculty of Science and CEITEC, Central European Institute of Technology, Masaryk University Brno, Kamenice 5, 625 00 Brno, Czech Republic; Faculty of Informatics, Masaryk University Brno, Botanická 68a, 602 00 Brno, Czech Republic; Institute of Computer Science, Masaryk University Brno, Botanická 68a, 602 00 Brno, Czech Republic; Skaggs School of Pharmacy and Pharmaceutical Sciences, University of California, 9500 Gilman Drive, San Diego, MC 0657 USA

**Keywords:** Partial atomic charges, Electronegativity Equalization Method, EEM, Quantum mechanics, QM, Drug-like molecules

## Abstract

**Background:**

Partial atomic charges describe the distribution of electron density in a molecule and therefore provide clues to the chemical behaviour of molecules. Recently, these charges have become popular in chemoinformatics, as they are informative descriptors that can be utilised in pharmacophore design, virtual screening, similarity searches etc. Especially conformationally-dependent charges perform very successfully. In particular, their fast and accurate calculation via the Electronegativity Equalization Method (EEM) seems very promising for chemoinformatics applications. Unfortunately, published EEM parameter sets include only parameters for basic atom types and they often miss parameters for halogens, phosphorus, sulphur, triple bonded carbon etc. Therefore their applicability for drug-like molecules is limited.

**Results:**

We have prepared six EEM parameter sets which enable the user to calculate EEM charges in a quality comparable to quantum mechanics (QM) charges based on the most common charge calculation schemes (i.e., MPA, NPA and AIM) and a robust QM approach (HF/6-311G, B3LYP/6-311G). The calculated EEM parameters exhibited very good quality on a training set ($R^2 > 0.9$) and also on a test set ($R^2 > 0.93$). They are applicable for at least 95 % of molecules in key drug databases (DrugBank, ChEMBL, Pubchem and ZINC) compared to less than 60 % of the molecules from these databases for which currently used EEM parameters are applicable.

**Conclusions:**

We developed EEM parameters enabling the fast calculation of high-quality partial atomic charges for almost all drug-like molecules. In parallel, we provide a software solution for their easy computation (http://ncbr.muni.cz/eem_parameters). It enables the direct application of EEM in chemoinformatics.

**Electronic supplementary material:**

The online version of this article (doi:10.1186/s13321-015-0107-1) contains supplementary material, which is available to authorized users.

## Background

Partial atomic charges are real numbers describing the distribution of electron density in a molecule, thus providing clues as to the chemical behaviour of molecules. The concept of charges began to be used in physical chemistry and organic chemistry. Afterwards, partial atomic charges were adopted by computational chemistry and molecular modelling, where they serve for calculating electrostatic interactions, describe the reactivity of the molecule etc. Specifically, they are applied in molecular dynamics, docking, conformational searches, binding site predictions etc. Recently, partial atomic charges also became popular in chemoinformatics, as they proved to be informative descriptors for QSAR and QSPR modelling [1–9] and for other applications [10–12]; they can be utilised in pharmacophore design [13–15], virtual screening [16–18], similarity searches [19–21], molecular structure comparison [22–24] etc.

The partial atomic charges cannot be determined experimentally or derived straightforwardly from the results of quantum mechanics (QM), and many different methods have been developed for their calculation. The most common method for charge calculation is an application of the QM approach and afterwards the utilisation of a charge calculation scheme. Charge calculation schemes can be based on orbital-based population analysis, on wave-function-dependent physical observables or on reproducing charge-dependent observables. Examples of orbital-based population analyses are Mulliken population analysis (MPA) [25, 26], Löwdin population analysis [27] and Natural population analysis (NPA) [28, 29]. Wave-function-dependent physical observables are used in the atoms-in-molecules (AIM) approach [30, 31], Hirshfeld population analysis [32–34], CHELPG [35] and Merz-Singh-Kollman (MK) [36, 37] method. The reproduction of charge-dependent observables is applied in the CM1, CM2, CM3, CM4, and CM5 approaches [38, 39].

Unfortunately, QM charge calculation approaches are very time-consuming. A markedly faster alternative is to employ empirical charge calculation approaches, which can also provide high-quality charges. These approaches can be divided into conformationally-independent, which are based on 2D structure (e.g., Gasteiger’s and Marsili’s PEOE [40, 41], GDAC [42], KCM [43], DENR [44]) and conformationally-dependent, calculated from 3D structure (e.g., EEM [45], QEq [46] or SQE [47, 48]). We would like to highlight that conformationally-dependent charges are considered to be more suitable for chemoinformatics applications [1–3, 7, 12, 20]. The reason is that these charges contain extensive information not only about chemical surrounding of atoms, i.e., its topology (2D structure based charges) but also geometry and “chemical quality” of the surrounding. Such information is missing, for example, in force field charges which use averaged atomic charges from large sets of structures. Therefore we only focus on conformationally-dependent atomic charges.

Electronegativity equalization method (EEM) is the most frequently used conformationally-dependent empirical charge calculation approach. It calculates charges using the following system of linear equations:

1 $$\begin{aligned} \left( \begin{array}{ccccc} B_1 &{} \frac{\kappa }{R_{1,2}} &{} \cdots &{} \frac{\kappa }{R_{1,N}} &{} -1 \\ \frac{\kappa }{R_{2,1}} &{} B_2 &{} \cdots &{} \frac{\kappa }{R_{2,N}} &{} -1 \\ \vdots &{} \vdots &{} \ddots &{} \vdots &{} \vdots \\ \frac{\kappa }{R_{N,1}} &{} \frac{\kappa }{R_{N,2}} &{} \cdots &{} B_N &{} -1 \\ 1 &{} 1 &{} \cdots &{} 1 &{} 0 \end{array} \right) \cdot \left( \begin{array}{c} q_1 \\ q_2 \\ \vdots \\ q_N \\ \bar{\chi }\end{array} \right) = \left( \begin{array}{c} -A_1 \\ -A_2 \\ \vdots \\ -A_N \\ Q \end{array} \right) \end{aligned}$$

where $q_i$ is the charge of an atom *i*; $R_{i,j}$ is the distance between atoms *i* and *j*; *Q* is the total charge of the molecule; *N* is the number of atoms in the molecule; $\kappa$ is the molecular electronegativity, and $A_i$, $B_i$ and $\kappa$ are empirical parameters. The parameters $A_i$ and $B_i$ vary for individual atom types, where atom type is a combination of element type and maximal bond order of the atom *i*. For example, atom type C2 means that the atom is carbon and it creates at least one double bond with its neighbors. An atom X in the aromatic ring is therefore also included into X2 atom type. The parameters $A_i$, $B_i$ and $\kappa$ are molecule independent and they are calculated from QM atomic charges by a process of EEM parameterization [49]. EEM is not only a fast charge calculation approach, but it can also provide highly accurate charges, i.e., they can mimic the QM charges for which EEM has been parameterized. On the other hand, EEM charges can be outperformed in certain situations. Specifically, QEq showed better agreement with experimental dipole moments [46] and SQE is presented as an extension of the EEM to obtain the correct size-dependence of the molecular polarizability [47]. But this drawback is compensated by a fact that the quality of EEM charges was documented by many successful applications [2, 3, 50–55] and they are clearly the most cited empirical conformationally-dependent charges.

Therefore, many EEM parameter sets for various QM charge calculation approaches were published later or recently (see Table 1). In parallel, a few freely available software tools also include an EEM charge calculation method (see Table 2).

**Table 1:** Summary information about published EEM parameters evaluated in this study

QM theory Level + basis set	Charge calc. scheme	EEM parameter set name	Published by	Elements and bond orders included$^\dag$
HF/STO-3G	MPA	Baek1991	Baekelandt et al. [56]	C, O, N, H, P, Al, Si
		Svob2007_cbeg2	Svobodova et al. [49]	C1, C2, O, N1, N2, H, S1
		Svob2007_cmet2	Svobodova et al. [49]	C1, C2, O, N1, N2, H, S1, Fe, Zn
		Svob2007_chal2	Svobodova et al. [49]	C1, C2, O, N1, N2, H, S1, Br, Cl, F, I
		Svob2007_hm2	Svobodova et al. [49]	C1, C2, O, N1, N2, H, S1, F, Cl, Br, I, Fe, Zn
HF/6-31G*	MK	Jir2008_hf	Jirouskova et al. [57]	C1, C2, O, N1, N2, H, S1, F, Cl, Br, Zn
B3LYP/6-31G*	MPA	Bult2002_mpa	Bultinck et al. [58]	C, O, N, H, F
	NPA	Bult2002_npa	Bultinck et al. [58]	C, O, N, H, F
		Ouy2009$^\ddag$	Ouyang et al. [59]	C, O, N, H
		Ouy2009_elem	Ouyang et al. [59]	C, O, N, H
	Hir.	Bult2002_hir	Bultinck et al. [58]	C, O, N, H, F
	MK	Bult2002_mk	Bultinck et al. [58]	C, O, N, H, F
		Jir2008_mk	Jirouskova et al. [57]	C1, C2, O, N1, N2, H, S1, F, Cl, Br, Zn
	CHELPG	Bult2002_che	Bultinck et al. [58]	C, O, N, H, F
	AIM	Bult2004_aim	Bultinck et al. [60]	C, O, N, H, F

^†^An element symbol with no further information (e.g., C) means that the EEM parameters are available for this element bound by all possible bond orders. The element symbol followed by a number (e.g., C1) means that the EEM parameters are only available for this element bound by a bond with an order described using this number

^‡^For this parameter set, C1 represents sp$^3$ hybridization, C2 sp$^2$ hybridization, C3 sp hybridization, etc.

**Table 2:** Information about freely available software tools enabling EEM charge calculation

Software	EEM parameters used by a software
OpenBabel [61]	It contains the embedded EEM parameter set Bult2002_mpa, which was parameterized for B3LYP/6-31G*/MPA charges. It does not allow any other EEM parameter set to be used
Balloon [23]	It contains an embedded EEM parameter set published by Puranen et al. [62], which was calculated by fitting to the MEP field. Balloon’s developers claim that the EEM charges calculated via Balloon should be comparable to B3LYP/cc-pVTZ/MPA. It does not allow any other EEM parameter set to be used
EEM SOLVER [63]	It allows the use of any input EEM parameter sets provided by the user. It does not contain any embedded EEM parameter sets

EEM recently began to be also used in chemoinformatics, giving very promising results [1–3, 64, 65]. Because of their rapid calculation, they can be easily computed for large sets of molecules (e.g., drug-like compounds). Unfortunately, a broader utilisation of EEM charges in chemoinformatics is now limited by the fact that available EEM parameter sets can only cover part of common organic molecules, as they only contain the parameters for some elements and certain bond orders (Table 1). For the above reasons, our aim with this work is to provide EEM parameter sets that cover most of the drug-like molecules and with accuracy comparable to QM charges. Specifically, we have parameterized EEM for frequently used charge calculation schemes, high enough QM theory levels and a large basis set. Afterwards, we compared the coverage and quality of our EEM parameter sets with previously published EEM parameter sets (see Table 1) and with EEM parameter sets embedded in software tools (see Table 2). Additionally, we have prepared a software solution, enabling the user to easily calculate EEM charges via our EEM parameters.

**Fig. 1:**
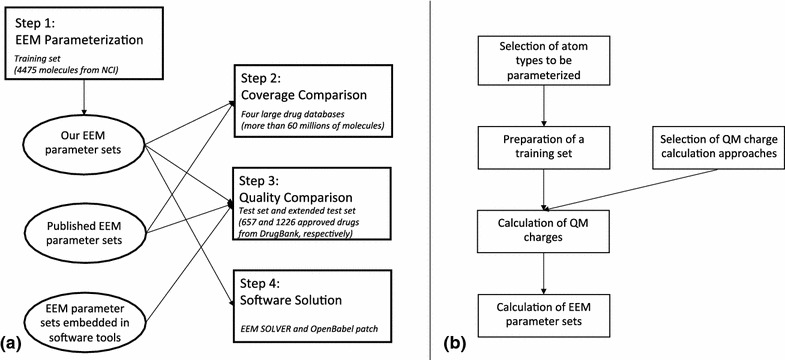
**a** Composition of steps performed within this work and **b** tasks performed during EEM parametrization

## Methods

### EEM parameterization (step 1)

All the steps performed during our work are depicted in Fig. 1a. The most challenging part of our work was the EEM parameterization. This step required several tasks (see Fig. 1b) and the quality of the calculated EEM parameters sets depends on the proper accomplishment of all these tasks.

#### EEM parameterization: selection of atom types to be parameterized

Our goal is to provide EEM parameter sets applicable for most common drug-like molecules. Therefore, we provide EEM parameters for the majority of atom types occurring in these molecules. These atom types are summarized in Table 3 (columns 1–3).

**Table 3:** Occurrence of atom types in the training set

Denotation of atom type	Element symbol	Maximal bond order	Number of atoms with this atom type in the training set	Number of molecules containing this atom type in the training set
H1	H	1	57,119	4442
C1	C	1	15,220	3447
C2		2	38,097	4149
C3		3	345	266
N1	N	1	4151	2483
N2		2	3383	1879
N3		3	345	266
O1	O	1	5016	2525
O2		2	5793	3069
F1	F	1	938	395
P1	P	1	153	143
P2		2	251	213
S1	S	1	1034	770
S2		2	1391	1211
Cl1	Cl	1	1084	676
Br1	Br	1	336	261
I1	I	1	1734	1365
Total	–	–	136,390	4475

#### EEM parameterization: preparation of the training set

Our training set contains the 3D structures of 4475 distinct small organic molecules. The molecules were obtained from the DTP NCI database [66] and their 3D structures were generated with CORINA 3.60 [67], without any further geometry optimization. The DTP NCI database collects compounds tested as anticancer drugs (with positive or negative results), therefore it is a database of common drug-like molecules. The training set was created in such a way that each selected atom type is contained in at least 100 molecules. The occurrences of individual atom types in the training set are summarized in Table 3. The list of training set molecules, including their NSC numbers and summary formulas, can be found in (Additional file 1: Table S1).

#### EEM parameterization: selection of QM charge calculation approach

We performed the EEM parameterization for two QM theory levels (B3LYP and HF), one basis set (6-311G) and three charge calculation schemes (MPA, NPA and AIM). We provide the EEM parameters for all combinations of these theory levels, the basis sets and the charge calculation schemes (see Table 4). Theory levels HF and B3LYP were selected, because they are very often used for QM charge calculation and were also successfully used for EEM parameterization several times [49, 56–60]. The basis set 6-311G was used, because it is robust, also covers iodine and moreover, Pople basis sets are very suitable for EEM parameterization. MPA and NPA population analyses were employed, because they are the most known charge calculation schemes and additionally, EEM is able to mimic MPA and NPA charges very successfully [49, 58, 59]. AIM was selected, because it is based on a different principle from the other two, and EEM can also mimic AIM charges very efficiently [60]. Note that we do not provide EEM parameters for ESP and RESP charges, because it is known that EEM does not mimic these charges well [2, 58].

**Table 4:** Quality criteria of our EEM parameter sets

EEM parameter set name	Relevant QM charges	R^2^	RMSD	${{\bar{\Delta }}}$
Cheminf_b3lyp_mpa	B3LYP/6-311G/MPA	0.9007	0.1038	0.0727
Cheminf_b3lyp_npa	B3LYP/6-311G/NPA	0.9651	0.0746	0.0540
Cheminf_b3lyp_aim	B3LYP/6-311G/AIM	0.9499	0.0785	0.0558
Cheminf_hf_mpa	HF/6-311G/MPA	0.9178	0.1125	0.0776
Cheminf_hf_npa	HF/6-311G/NPA	0.9633	0.0805	0.0574
Cheminf_hf_aim	HF/6-311G/AIM	0.9441	0.0919	0.0651

#### EEM parameterization: calculation of QM charges

For each molecule from the training set, six sets of QM charges were calculated via the above-mentioned six QM charge calculation approaches. The calculations of QM charges were carried out using Gaussian09 [68]. With the AIM population analysis, the output from Gaussian03 was further processed with the software package AIMAll [69].

#### EEM parameterization: calculation of EEM parameter sets

For each set of QM charges, the EEM parameterization was performed and the values of the parameters are provided in (Additional file 2: EEM parameters). The software NEEMP [70] was used for the parameterization. This software implements the parameterization methodology described by [49] and introduces several marked improvements into it. NEEMP provides EEM parameter sets together with their quality criteria, i.e., squared Pearson correlation coefficient ($R^2$), root mean square deviation (RMSD), and average absolute error (${\overline{\Delta }}$), calculated via Eqs. (2), (3) and (4), respectively

2 $$\begin{aligned} R^2 = \frac{\left(\sum \limits _{i=1}^{N}\left( q^{EEM}_i - \overline{q}^{EEM}\right) \left( q^{QM}_i - \overline{q}^{QM}\right)\right)^2}{\sum \limits _{i=1}^{N}\left( q^{EEM}_i - \overline{q}^{EEM}\right) ^2 \sum \limits _{i=1}^{N}\left( q^{QM}_i - \overline{q}^{QM}\right) ^2} \end{aligned}$$

3 $$\begin{aligned} \mathrm {RMSD} = \sqrt{ \frac{\sum \limits _{i=1}^{N}\left( q^{EEM}_i - q^{QM}_i\right) ^2}{N} } \end{aligned}$$

4 $$\begin{aligned} {\overline{\Delta }} = \frac{\sum \limits _{i=1}^{N}\left| q^{EEM}_i - q^{QM}_i \right| }{N} \end{aligned}$$

where $q^{EEM}_i$ is the EEM charge of an atom *i*; $q^{QM}_i$ is the QM charge of an atom *i*; $\overline{q}^{EEM}$ is an average of all EEM charges; $\overline{q}^{QM}$ is an average of all QM charges, *N* is the number of atoms in the molecule.

### Coverage comparison (step 2)

For comparison, we used our six EEM parameter sets and 15 published EEM parameter sets, described in Table 1 (all 21 of these EEM parameter sets will be below referred to as the tested EEM parameter sets). The coverage comparison was done on four very well-known databases of drug-like chemical compounds: DrugBank [71, 72], ChEMBL [73], PubChem [74], and ZINC [75]. The number of compounds in all these databases (from 10$\mathrm {^{th}}$ February 2015) are summarized in Table 5. For each tested EEM parameter set, we analysed how many compounds from the four databases can be covered by them (i.e., contains only atom types present in the tested EEM parameter sets). This coverage analysis was done using NEEMP.

**Table 5:** Size of database, used for comparison of EEM parameter set coverages

Database	Number of compounds
DrugBank	6874
ChEMBL	1,456,020
PubChem	63,676,639
ZINC	21,957,378

### Quality comparison (step 3)

This evaluation was done for the 21 above-mentioned tested EEM parameter sets and was performed on two data sets—a test set (657 molecules) and an extended test set (1226 molecules). The extended test set contained all approved drugs (i.e., drugs which have received approval in at least one country) from the DrugBank database (downloaded 10th February 2015), for which it was possible to calculate all QM charges necessary for testing. The test set was a subset of the extended test set, which contained only molecules covered by all the tested EEM parameter sets. The 2D structures of all molecules were obtained from DrugBank. The lists of molecules from the test set and the extended test set, including their DrugBank IDs and summary formulas, can be found in (Additional file 3: Table S2a; Additional file 4: Table S2b, respectively). The 3D structures of all the molecules were generated with CORINA 2.6 [67], without any further geometry optimization. For all the molecules, we calculated all the types of QM charges which corresponded to the tested EEM parameters. This means we used the 8 QM charge calculation approaches mentioned in Table 1 and the six QM charge calculation approaches employed for calculating our EEM parameter sets. The calculations of QM charges were done with Gaussian09 and the AIMAll software package was used for AIM charges. We compared the quality of the tested EEM parameter set on both the test set and the extended test set. The comparison was done using NEEMP, which provided quality criteria for all the tested EEM parameter sets. In the extended test set, some molecules were not covered by certain EEM parameter set(s). Therefore, we calculated quality criteria based purely on the covered molecules and in parallel, we also computed the coverage.

#### Quality comparison: EEM parameter sets embedded in software tools

The calculation of EEM charges can be done with a few software tools, e.g., EEM SOLVER, OpenBabel or Balloon. The software tools OpenBabel and Balloon contain embedded EEM parameter sets (see Table 2). Therefore, we also evaluated the quality of these embedded EEM parameter sets. This evaluation was done for the same data sets and via the same procedure as with the tested EEM parameter sets. The only difference was that the EEM charges were not calculated with NEEMP, but with OpenBabel and Balloon. Afterwards, these EEM charges were compared with the relevant QM charges using R statistical software [76], which provided their quality criteria.

### Software solution (step 4)

We provide the user two such solutions, the first based on EEM SOLVER and the second on OpenBabel.

## Results and discussion

### EEM parameterization (step 1)

EEM parameterization was performed for six QM charge calculation approaches, and a training set containing 4475 drug-like molecules was used. Squared Pearson correlation coefficient ($R^2$), root mean square deviation (RMSD) and average absolute error (${\overline{\Delta }}$) of the obtained EEM parameter sets, calculated for the training set, are summarized in Table 4. These quality criteria describe the correlation between QM charges and the corresponding EEM charges and they were calculated using NEEMP software.

These results show that the quality of our EEM parameter sets is very high, i.e., all the $R^2$ values are higher or equal to 0.9. Table 4 also illustrates that QM theory levels B3LYP and HF are both applicable for EEM parameterization, and EEM charges based on them have similar accuracy. From this table, we can also see that the quality of EEM parameters based on NPA and AIM population analysis is slightly better than for MPA.

### Coverage comparison (step 2)

Information about the coverages of published EEM parameter sets and our EEM parameter sets are summarized in Table 6. The coverages were computed on four well-known databases of drug-like molecules—DrugBank, ChEMBL, PubChem and ZINC. Table 6 shows that the coverages of the published EEM parameter sets are low ($<$60 %). The only exception are the EEM parameter sets published by Svobodova et al. and Jirouskova et al., which have coverage between 70 and 80 %. In contrast, our EEM parameter sets have very high coverage—about 95 % or more for all the databases. The not covered molecules include atom types rare for drug-like molecules, e.g., metals or boron. An interesting fact is that the coverages are very similar for all four analyzed databases. Therefore, low EEM parameter set coverage is not merely an isolated issue related to one database, but a general problem.

### Quality comparison (step 3)

Table 6 summarizes the main quality criteria (i.e., $R^2$ values) of all tested EEM parameter sets for the test set, which contained 657 approved drugs from DrugBank. Other quality criteria (RMSD and ${\overline{\Delta }}$) can be found in (Additional file 5: Table S3) and all values of partial atomic charges (represented as tables and as graphs) are in (Additional file 6). The table shows that our EEM parameter sets are among the best performing EEM parameter sets to have been published so far. The table also illustrates that the quality of EEM parameters is strongly influenced by the selection of QM charge calculation scheme. Specifically, EEM parameters based on MPA, NPA and AIM charges are very high quality, and EEM parameters based on Hirshfeld charges are still acceptable. EEM parameters based on MK and CHELPG charges are very low quality, which is in agreement with published data [2, 58]. Both theory levels (HF and B3LYP) and all three basis sets used (STO-3G, 6-31G* and 6-311G) are applicable for EEM parameterization. These results also confirm that our selection of QM theory level, basis set and charge calculation schemes is appropriate.

For the extended test set, the quality criteria exhibit similar trends (see Additional file 7: Table S4). In parallel, the coverages for this data set are slightly higher than for the complete DrugBank database. An interesting fact is that even for such common compounds as approved drugs, the coverages of published EEM parameter sets are low. Specifically, most published EEM parameter sets have coverages between 55 and 65 %. Further remarkable fact is that quality criteria of our EEM parameters are better for the test set than for the training set. The reason is that the training set is much larger and heterogeneous than the test set.

#### Quality comparison: EEM parameter sets embedded in software tools

EEM charges produced with OpenBabel were compared with QM charges calculated with B3LYP/6-31G*/MPA. The quality criteria for the test set were the same as for the EEM parameters Bult2002_mpa (i.e., $R^2$ about 0.97). This was expected, because OpenBabel uses Bult2002_mpa as its embedded EEM parameters. Very surprising was the behavior of OpenBabel on the extended set. The coverage was 100 %, but the quality criteria were markedly lower (e.g., $R^2$ about 0.82). The reason for this is that OpenBabel replaces the EEM parameters for atom types which are not provided in Bult2002_mpa with the EEM parameters for some other atom types. Unfortunately, this approach is not very reliable, i.e., the quality criteria for molecules which are in the extended test set but are not in the test set are very low ($R^2 = 0.66$). Additionally, this approach is relatively tricky. The user does not know whether the correct or the estimated EEM parameters are used and, therefore, whether the resulting EEM charges will be of a good quality.

The EEM charges produced by Balloon were compared with the QM charges calculated by the B3LYP/cc-pVTZ/MPA approach. The coverage was close to 100 %, but the correlation was also low ($R^2 < 0.8$). On the other hand, the Balloon developers mentioned that the EEM charges provided by Balloon do not correspond directly to some particular QM charges, and they should only be close to B3LYP/cc-pVTZ/MPA charges.

All the quality criteria and coverages for EEM parameter sets embedded in OpenBabel and Balloon are summarized in (Additional file 8: Table S5).

### Coverage comparison and quality comparison combined

To date, there have been no EEM parameter sets available which would provide both high coverage and high-quality EEM charges (see Table 6). On the other hand, the EEM parameter sets calculated in this paper solve this problem, because they exhibit coverage close to 100 % and excellent quality criteria. Therefore, they can be used for chemoinformatics applications.

**Table 6:**
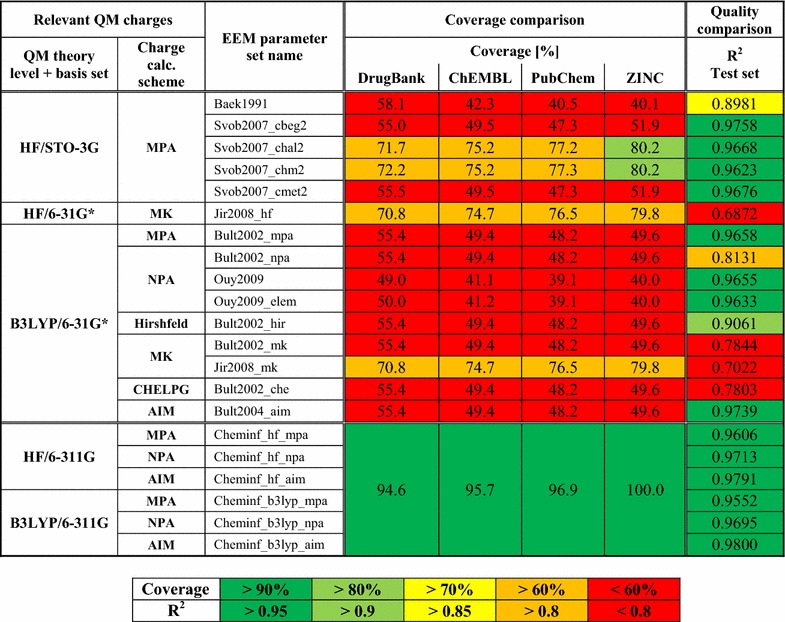
Summary information about coverage and quality of all tested EEM parameters (see below for meaning of colours)

### Software solution (step 4)

For the actual applicability of EEM in chemoinformatics, the user doesn’t just need EEM parameter sets that are high quality and cover almost all molecules. They also need a software package that embeds these EEM parameter sets and calculates EEM charges based on them. We provide the user with two such solutions. First, we provide our EEM parameter sets in a format that can be directly used in EEM SOLVER (Additional file 2: EEM parameter sets). Second, we provide an OpenBabel patch which allows our EEM parameter sets to be used directly in OpenBabel (Additional file 9: OpenBabel patch). All the information including documentation is also accessible on the web: http://ncbr.muni.cz/eem_parameters. The parameters are also accessible via ACC web application [77].

## Conclusion

We provide here six EEM parameter sets which enable the user to calculate EEM charges with quality comparable to frequently used QM charges computed by well-known charge calculation schemes (i.e., MPA, NPA and AIM) and based on a robust QM approach (HF/6-311G, B3LYP/6-311G). The training set for EEM parameterization contained more than 4000 molecules from the DTP NCI drug database, and all six calculated EEM parameter sets exhibited a very good quality on this training set ($R^2 > 0.9$).

The coverage of these computed EEM parameter sets was then compared with the coverages of 15 EEM parameter sets published in the past. This comparison was done on four key databases of drug-like molecules—DrugBank, ChEMBL, Pubchem and ZINC. The comparison showed that our EEM parameter sets enable us to calculate EEM charges for almost all molecules in these databases.

We then compared the quality of computed and published EEM parameter sets on two test data sets composed of approved drugs from DrugBank. This comparison also included EEM parameter sets embedded in the software tools OpenBabel and Balloon. The comparison showed that our EEM parameter sets are among the best performing EEM parameter sets published to date ($R^2 > 0.93$).

To summarize, charge calculation methodology suitable for chemoinformatics applications like virtual screening or QSAR should be fast, conformationally-dependent and accurate. EEM fulfils all these requirements. However, EEM parameter sets that would exhibit high coverage of drug-like molecule databases and provide high quality charges have not been available to date. The EEM parameters calculated in this paper solve this problem. They exhibit coverage close to 100 % and excellent quality criteria, therefore they are applicable in chemoinformatics.

Last but not least, we provide a software solution for the easy computing of EEM charges based on these EEM parameter sets—input files for EEM SOLVER and OpenBabel patch.

## Additional files

10.1186/s13321-015-0107-1 List of training set molecules, including their NSC numbers and summary formulas.
10.1186/s13321-015-0107-1 EEM parameters. Values of EEM parameter sets for these six charge calculation approaches (i.e. B3LYP/6-311G/MPA,B3LYP/6-311G/NPA, B3LYP/6-311G/AIM, HF/6-311G/MPA, HF/6-311G/NPA, and HF/6-311G/AIM). These EEM parameter sets are in a format which can be used as an input file for EEM SOLVER.
10.1186/s13321-015-0107-1 A list of molecules from the test set including their DrugBank IDs and summary formulas.
10.1186/s13321-015-0107-1 A list of molecules from the extended test set including their DrugBank IDs and summary formulas.
10.1186/s13321-015-0107-1 RMSD and ${\overline{\Delta }}$ values of all tested EEM parameter sets on the test set.
10.1186/s13321-015-0107-1 Charge details. Values of partial atomic charges (represented as tables and as graphs) for all tested EEM parameter sets on the testset.
10.1186/s13321-015-0107-1 *R* ^2^, RMSD, ${\overline{\Delta }}$ and coverage values of all tested EEM parameter sets on the extended test set.
10.1186/s13321-015-0107-1 RMSD and ${\overline{\Delta }}$ values for OpenBabel and Balloon on the test set and extended test set.
10.1186/s13321-015-0107-1 OpenBabel patch. A patch for OpenBabel, which enables it to use the EEM parameter sets calculated in this paper.
